# An Alzheimer’s Disease Genetic Risk Score Predicts Longitudinal Thinning of Hippocampal Complex Subregions in Healthy Older Adults

**DOI:** 10.1523/ENEURO.0098-16.2016

**Published:** 2016-07-15

**Authors:** Theresa M. Harrison, Zanjbeel Mahmood, Edward P. Lau, Alexandra M. Karacozoff, Alison C. Burggren, Gary W. Small, Susan Y. Bookheimer

**Affiliations:** 1 Neuroscience Interdepartmental Graduate Program, University of California, Los Angeles, Los Angeles, California 90095; 2 Department of Psychiatry and Biobehavioral Sciences, University of California, Los Angeles, Los Angeles, California 90095; 3 Staglin IMHRO Center for Cognitive Neuroscience, University of California, Los Angeles, Los Angeles, California 90095; 4 Semel Institute for Neuroscience and Human Behaviors, University of California, Los Angeles, Los Angeles, California 90095; 5 UCLA Longevity Center, University of California, Los Angeles, Los Angeles, California 90095

**Keywords:** clinical trials, normal aging, polygenic risk score, preclinical Alzheimer's disease, structural MRI

## Abstract

Variants at 21 genetic loci have been associated with an increased risk for Alzheimer’s disease (AD). An important unresolved question is whether multiple genetic risk factors can be combined to increase the power to detect changes in neuroimaging biomarkers for AD. We acquired high-resolution structural images of the hippocampus in 66 healthy, older human subjects. For 45 of these subjects, longitudinal 2-year follow-up data were also available. We calculated an additive AD genetic risk score for each participant and contrasted this with a weighted risk score (WRS) approach. Each score included *APOE* (apolipoprotein E), *CLU* (clusterin), *PICALM* (phosphatidylinositol binding clathrin assembly protein), and family history of AD. Both unweighted risk score (URS) and WRS correlated strongly with the percentage change in thickness across the whole hippocampal complex (URS: *r* = −0.40; *p* = 0.003; WRS: *r* = −0.25, *p* = 0.048), driven by a strong relationship to entorhinal cortex thinning (URS: *r* = −0.35; *p* = 0.009; WRS: *r* = −0.35, *p* = 0.009). By contrast, at baseline the risk scores showed no relationship to thickness in any hippocampal complex subregion. These results provide compelling evidence that polygenic AD risk scores may be especially sensitive to structural change over time in regions affected early in AD, like the hippocampus and adjacent entorhinal cortex. This work also supports the paradigm of studying genetic risk for disease in healthy volunteers. Together, these findings will inform clinical trial design by supporting the idea that genetic prescreening in healthy control subjects can be useful to maximize the ability to detect an effect on a longitudinal neuroimaging endpoint, like hippocampal complex cortical thickness.

## Significance Statement

This is the first study to show a relationship between a genetic risk score (GRS) for Alzheimer’s disease (AD) and hippocampal thinning in healthy adults. We found that a GRS composed of AD risk factors that have been shown to relate to hippocampal structure or function in humans predicted thinning of the hippocampal complex. Our ability to interpret these findings is bolstered by the association of genetic risk with longitudinal atrophy as opposed to cross-sectional morphology, which might be driven by neurodevelopmental differences. This work has implications for clinical trials focused on preclinical subjects such that screening by polygenic risk might increase the ability to detect an effect of a drug in a trial where hippocampal integrity is an endpoint.

## Introduction

The development of preclinical biomarkers for sporadic, late-onset Alzheimer’s disease (AD) is critical for clinical trial design, and ultimately for disease prevention. Neuronal loss in the hippocampus occurs early in the course of AD. This neuronal loss leads to morphological changes over time resulting in severe atrophy of the entire hippocampus in advanced AD. The hippocampus, however, begins to shrink long before the emergence of clinical symptoms. Research on families who carry genetic mutations for dominantly inherited AD has revealed that hippocampal volume loss is detectable up to 15 years before the expected onset of symptoms (Bateman et al., 2012). Studies have shown that a key difference between normal age-related hippocampal thinning and pathological thinning related to AD may be the rate of thinning over time (Lu et al., 2011; Chincarini et al., 2016). Longitudinal data are, therefore, extremely important in predicting the trajectories of normal and pathological aging.

Genetic risk for AD is also related to hippocampal thinning. Carriage of the apolipoprotein E ε4 allele (APOEε4) allele accelerates age-related hippocampal shrinkage in older healthy adults, which may make individuals more susceptible to AD (Jak et al., 2007; Donix et al., 2010a). While *APOE* is the strongest genetic risk factor for AD, at least 20 other genes have been identified as being associated with the disease (Lambert et al., 2013). Among these non-*APOE* AD risk genes, clusterin (*CLU*) and phosphatidylinositol binding clathrin assembly protein (*PICALM*) have been studied using a neuroimaging genetics approach more than any other risk genes (Biffi et al., 2010; Bralten et al., 2011; Braskie et al., 2011; Erk et al., 2011; Furney et al., 2011; Hohman et al., 2013; Green et al., 2014; Stevens et al., 2014; Harrison et al., 2015; Zhang et al., 2015; Harrison and Bookheimer, 2016). Also, a family history of AD can serve as a proxy for genetic risk and has been used in neuroimaging genetics studies to identify characteristics of a high-risk group (Xu et al., 2009; Berti et al., 2011; Honea et al., 2012; Wang et al., 2012). Each of these factors, *APOE*, *CLU*, *PICALM*, and a family history of AD, has been previously shown to be related to hippocampal structure or function, as measured by MRI-based techniques in humans (Biffi et al., 2010; Donix et al., 2010b; Erk et al., 2011; Furney et al., 2011; Honea et al., 2011; Okonkwo et al., 2012; Zhang et al., 2015). Thus, we selected these components to calculate a genetic risk score (GRS) based on their statistical association with AD risk and their previous association with the hippocampus in neuroimaging genetics studies.

The use of high-resolution structural MRI to calculate the thickness of the strip of gray matter within the convoluted hippocampus allows for the sensitive measurement of changes in morphology (Ekstrom et al., 2009). This approach is preferable to measuring the gross volume of the hippocampus because it focuses on the compartment of the hippocampal complex where cell bodies reside and thus is designed to measure morphological changes that may be related to neuronal loss. Using hippocampal thickness measurements, subregions of the hippocampal complex, including entorhinal cortex, subiculum, CA3, and dentate gyrus, have been shown to be thinner in APOEε4 carriers compared to noncarriers (Burggren et al., 2008; Mueller et al., 2008; Mueller and Weiner, 2009). In this work, we take these findings further by expanding our focus to include additional genetic risk factors for AD.

The present study is the first to find evidence of an association of an AD GRS and cortical thinning of the hippocampus over time in healthy adults. By focusing our GRS development on genetic factors that have been shown to associate with hippocampal structure or function in healthy older adults, we were able to boost our power to detect a link between the genetic risk for AD and changes in hippocampal gray matter. Our findings support the validity of a neuroimaging genetics approach to studying genetic risk for disease in healthy, preclinical populations. Identifying quantitative neuroimaging endophenotypes associated with genetic risk for AD in healthy adults will increase our ability to identify healthy individuals who are at greatest risk for the development of AD and target them for intervention. In the present study, we hypothesized that the AD GRS would be related to baseline hippocampal morphology when controlling for confounding factors like age and sex. We further hypothesized that the GRS would predict longitudinal thinning in the hippocampal complex, especially the entorhinal cortex and subiculum subregions, over a 2 year follow-up period.

## Materials and Methods

### Participants

Participants for this study were Caucasian individuals of either sex recruited as part of an ongoing initiative to study aging, AD genetic risk, and dementia by the University of California, Los Angeles (UCLA) Longevity Center. The recruitment strategy focused on older adult community centers, relatives of AD patients referred by the local Alzheimer’s Association chapter, memory groups, and other groups catering to older adults with age-related memory concerns. This strategy resulted in the recruitment of ∼40–50% of participants carrying at least one copy of the APOEε4 allele, which is greater than the 20-25% that would be expected from purely random recruitment (Bookheimer et al., 2000; Small et al., 2000). Participants were categorized as having a positive family history of AD if at least one first-degree relative had received a diagnosis of AD based on standard criteria (Dubois et al., 2007). All participants in the present study were healthy and cognitively intact at the time of study enrollment. In our study, participants were defined as nondemented if they were cognitively intact based on clinical examination, the results of the mini mental state exam (MMSE; for gross cognition, threshold ≥27) and standard criteria for age-associated memory impairment; specifically, participants were excluded if they had scores >2 SDs below normal on two or more of the memory tests described in the next section. In addition, participants with clinical anxiety, depression, or any neuropsychiatric or neurological illness were excluded. This study was performed in accordance with UCLA Institutional Review Board protocols and was approved by the UCLA Human Subjects Protection Committee. All participants gave written informed consent upon enrollment in this study.

### Neuropsychological assessment

A 3 h neuropsychological battery was administered to each participant. The battery included tests of the following: general intelligence (subtests of the Wechsler Adult Intelligence Scale, third edition; Wechsler, 1997), fluency (Fruits and Vegetables; Cauthen, 1978), attention (Digits Forward and Backward; Wechsler, 1997), language (Boston Naming Test; Goodglass and Kaplan, 2001), verbal memory (Buschke-Fuld Selective Reminding Task; (Buschke and Fuld, 1974) as well as Wechsler Memory Scale, third edition, logical memory and verbal paired associates learning (Wechsler, 1997)), and visual memory (Rey-Osterrieth figure test; Osterrieth, 1944). Participants also completed a family history questionnaire (Breitner and Folstein, 1984), a memory complaints self-report questionnaire (Gilewski et al., 1990), the Hamilton Depression and Anxiety Inventories (Hamilton, 1959, 1960), the Neuropsychiatric Inventory (Cummings et al., 1994), and the MMSE (Folstein et al., 1983).

### Genotyping

A trained phlebotomist at the UCLA Clinical and Translational Research Laboratory drew a blood sample from each participant. Leukocytes from 10 ml of the sample were frozen and stored at −80°C. Two hundred micrograms of genomic DNA was isolated from the remaining 10 ml of the sample and were screened using a PCR-based mutation detection assay and microsatellite marker-based genotyping. Real-time PCR on an Applied Biosystems 7900HT Fast Real-Time PCR System was used to perform genotyping of *APOE* single nucleotide polymorphisms (SNP; rs429358 and rs7412). In addition to a standard curve amplification protocol, an allelic discrimination step was added to facilitate the contrast between the two alleles and their respective reporter dyes. These dyes are incorporated into a TaqMan SNP Genotyping Assay with identification numbers C_3084793_20 and C_904973_10 for rs429358 and rs7412, respectively (Applied Biosystems). Results were confirmed by repeating the experiment. SNP genotyping data were analyzed using SDS software (version 2.3, Applied Biosystems). This program calculates the affinity of the sample to one of the two reporter dyes that, in turn, represents one allele over the other. *CLU* (rs11136000) and *PICALM* (rs3851179) SNPs were genotyped using iPLEX chemistry on the massARRAY platform (Sequenom) as per the manufacturer instructions. The assay was based on primer extension and allowed for a locus-specific PCR followed by an extension reaction in which the primer anneals immediately upstream of the polymorphic site being genotyped. Through the use of matrix-assisted laser desorption/ionization time-of-flight mass spectrometry the mass of the extended primer is determined. Sequenom Typer software automatically translates the mass of the observed primers into a genotype. Positive controls were included on every chip to ensure genotyping accuracy. The results of all genotyping protocols are strictly confidential and are never revealed to the research participant.

### Genetic risk scores

**Figure 1.**
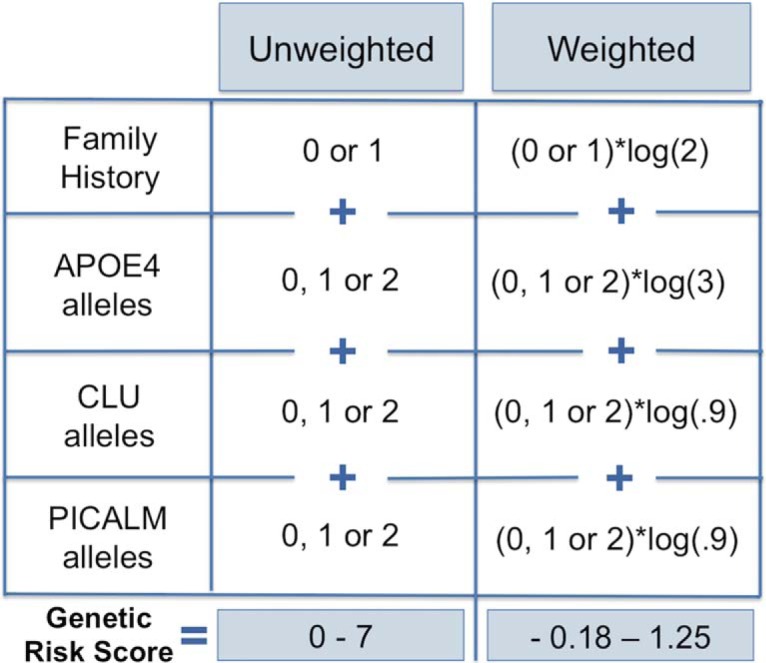
Genetic risk score calculation. A URS for each participant was calculated by adding family history of AD (0 if negative history or 1 if positive history), and the number of APOEε4 alleles (0, 1, or 2), *CLU* risk alleles (0, 1, or 2), and *PICALM* risk alleles (0, 1, or 2). A WRS for each participant was calculated using the logarithm of published ORs to weight the relative contribution of the factors before summing: positive family history (OR = 2), APOEε4 (OR = 3), *CLU* minor allele (OR = 0.9), PICALM minor allele (OR = 0.9). Possible unweighted risk scores range from 0 to 7, and weighted risk scores range from −0.18 to 1.25.

A GRS for AD was calculated for each participant. The GRS measured genetic risk load for AD across *APOE*, *CLU*, and *PICALM*, as well as taking into account the family history of AD. We calculated two sets of GRSs: unweighted risk score (URS) and weighted risk score (WRS). The URS was the sum of risk factors including a family history of AD (0 if negative history or 1 if positive history), APOEε4 alleles (0, 1, or 2), *CLU* risk alleles (0, 1, or 2) and *PICALM* risk alleles (0, 1, or 2; Fig. 1). For the WRSs, we used the logarithm of published odds ratios (ORs) to weight the relative contribution of these following risk factors before summing: positive family history, OR = 2; APOEε4, OR = 3; *CLU* minor allele, OR = 0.9; *PICALM* minor allele, OR = 0.9 (Lambert et al., 2013). We chose to focus our GRS on these risk factors because they are among the most consistently reproduced genetic risk factors associated with late-onset sporadic AD. In addition, each of these factors has been previously shown to be a related hippocampal structure or function, as measured using MRI-based techniques in humans (Biffi et al., 2010; Donix et al., 2010b; Erk et al., 2011; Furney et al., 2011; Honea et al., 2011; Okonkwo et al., 2012; Zhang et al., 2015).

### Imaging acquisition

MRI acquisition was completed using a Siemens 3 T Trio magnet located at the UCLA Staglin IMHRO Center for Cognitive Neuroscience (scans acquired 2010-2012; *n* = 8 baseline, *n* = 13 follow-up) or a Siemens Allegra 3 T scanner located at the UCLA Brain Mapping Center (scans acquired 2006–2010). Whole-brain 3D T1-weighted magnetization-prepared rapid acquisition gradient echo (MPRAGE) volumetric scans and high-resolution oblique coronal T2-weighted fast spin echo sequences were acquired with each participant. Scan parameters are as follows for the MPRAGE (parameters for the Allegra 3 T scanner are in parentheses): axial slicing; TR = 1900 ms (2300 ms); TE = 2.26 ms (2.93 ms); FOV = 250 × 218 mm (256 × 256 mm); flip angle = 9°; matrix = 256 × 215 mm; 176 slices (160 slices); slice thickness = 1 mm; zero-filled to a matrix of 256 × 224, resulting in a voxel size of 1 × 0.976 × 0.976 mm^3^ (1 × 1.3 × 1.3mm^3^). For the high-resolution hippocampal structural imaging, sequence parameters are as follows: TR = 5200 ms; TE = 107 ms (105 ms); FOV = 200 × 200 mm; flip angle = 139°; matrix = 512 × 512 mm; slice thickness = 3 mm; spacing, 0 mm; 19 slices; in-plane voxel size = 0.39 × 0.39 mm. Some participants’ whole-brain or high-resolution hippocampal structural imaging data have been used in previous publications (Donix et al., 2010a,b,2013; Brown et al., 2011; Burggren et al., 2011; Burggren and Brown, 2014).

**Figure 2.**
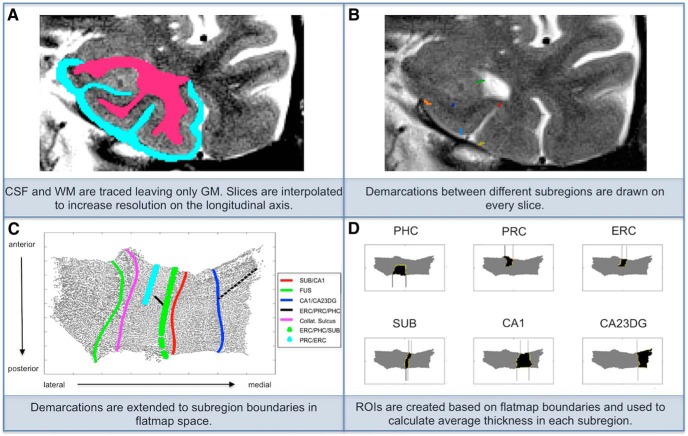
High-resolution hippocampal imaging processing steps. ***A***, Manual segmentation results in the following three distinct compartments: cerebrospinal fluid (CSF) (teal), white matter (WM) (pink), and gray matter (GM) in between. ***B***, The boundaries between hippocampal complex subregions are marked according to anatomical landmarks. Demarcations include CA23DG | CA1 (green), CA1 | subiculum (dark blue), subiculum | entorhinal cortex (orange), perhinal cortex | entorhinal (light blue), collateral sulcus (red), and fusiform gyrus (yellow). ***C***, These demarcations are extended along the longitudinal axis of the hippocampal complex to form complete and smooth boundaries between subregions. ***D***, Each subregion is then considered separately as a region of interest (ROI), and average thickness is calculated for each.

### Statistical and imaging analyses

#### Neuropsychological performance

To test whether participants in the baseline group differed from the subset with follow-up data, two-tailed *t* tests were used to examine age, sex, education, and general cognition. We examined potential relationships between genetic risk load and sex or age using a *t* test and Pearson correlation, respectively. These tests were completed using tools from the R Project for Statistical Computing (http://www.r-project.org; Research Resource Identifier, SCR_001905).

#### Whole-brain structural imaging

Whole-brain structural MRI scans were processed using Freesurfer (Fischl and Dale, 2000). This computational neuroanatomy software suite uses tissue contrast to determine the boundary between gray matter and white matter as well as the pial surface of the brain, and calculates the distance between vertices plotted as a mesh on each surface across the whole cortex. After completing the FreeSurfer automated pipeline, each participant’s scan was visually checked for accuracy. Minimal manual edits were completed when necessary by a single individual (T.M.H.). Intracranial volume (ICV) estimates from FreeSurfer were used to normalize hippocampal thickness estimates from baseline scans for baseline-only analyses. We used the following formula in order to normalize: ICV-corrected thickness = [(thickness in mm/ICV in mm^3^) * 10^6^]. Multiplying the quotient by 10^6^ results in values at the same order of magnitude as the original thickness estimates.

#### High-resolution hippocampal structural imaging

A cortical segmentation and unfolding procedure was used to measure the thickness of the gray matter of the hippocampal complex (HC) (Burggren et al., 2008, 2011; Ekstrom et al., 2009; Donix et al., 2010b; Fig. 2). First, the white matter and cerebrospinal fluid (CSF) within the medial temporal lobe were manually traced on oblique coronal slices. Slices were acquired from the scanner at intervals of 3 mm perpendicular to the longitudinal axis of the HC to maximize the resolution where anatomical variability is greatest. To account for slice thickness, a procedure that creates six linearly interpolated slices between each acquired pair was used to increase resolution along the longitudinal axis of the HC (Zeineh et al., 2001). The interpolation procedure resulted in a final voxel size of 0.39 × 0.39 × 0.43 mm (for two subjects, the final voxel size was 0.39 × 0.39 × 0.56 mm due to a thicker slice interval of 3.9 mm). Next, up to 18 contiguous layers of gray matter were created using a region-expansion algorithm starting at the white matter boundary and continuing to the CSF boundary. This results in an HC gray matter strip, which contains cornu ammonis (CA) fields 1, 2, and 3; the dentate gyrus (DG); the subiculum (SUB); entorhinal cortex (ERC); perirhinal cortex (PRC); parahippocampal cortex (PHC); and the fusiform gyrus (FUS). Our resolution is not high enough to reliably distinguish between DG and CA fields 2 and 3, so we combine these regions into a single subregion denoted CA23DG. Next, the gray matter strip was flattened using an iterative algorithm based on multidimensional scaling that has been used previously by our group (Ekstrom et al., 2009). Demarcations indicating the boundaries between different HC subregions were drawn on each slice based on anatomical landmarks in histological and MRI atlases (Amaral and Insausti, 1990; Mai and Paxinos, 1997; Duvernoy, 2005). Demarcations were placed in accordance with guidelines and findings produced by the Hippocampal Subfields Group (Yushkevich et al., 2015). The demarcations are extended to form continuous boundaries between subregions in 2D space. ROIs are drawn in 2D space and transformed back into in-plane space where gray matter thickness measurements were calculated. To calculate thickness, we computed the distance to the closest non-gray matter voxel. Specifically, in 2D space, for each middle point voxel, the maximum distance value for all of the corresponding 3D voxels across the layers of the gray matter strip was multiplied by 2. The mean thickness was calculated by averaging this value across all of the 2D voxels within a given subregion. We averaged each subregion across left and right hemispheres as we did not have any specific hypotheses regarding the laterality of an association between longitudinal change in hippocampal structure and genetic risk for AD.

Manual segmentations of baseline and follow-up scans for each participant were inspected by a single individual (Z.M.) to ensure consistency and minimize noise in our thickness estimates. During image processing, investigators were blinded to the demographic and genetic information corresponding to each image.

Associations between baseline thickness estimates corrected for ICV and the GRS were tested using Pearson correlations. To examine thinning over time, the percentage change in cortical thickness between baseline and follow-up scans was calculated for each participant with longitudinal data. The formula for calculating the percentage change was as follows: [((thickness at follow-up/thickness at baseline) − 1) * 100]. The percentage change statistics were not corrected for ICV, as measuring the percentage change in thickness within subjects obviates the need to control for normal variation in brain size. We also calculated partial correlations between GRS and baseline thickness or the percentage change in thickness controlling for the effects of age, sex, and time between visits, when appropriate.

Corrections for multiple comparisons were performed within each GRS because they were highly correlated and not independent (*r* = 0.72, *p* < 0.0001). We used a Bonferroni correction for two independent tests (*p* = 0.05/2 = 0.025) to control for multiple testing in entorhinal cortex and subiculum, the two regions in which we hypothesized that thinning would be related to genetic risk for AD. These tests were simple effects tests following whole HC analysis. Because entorhinal cortex and subiculum are subregions of the whole HC, these are not independent tests. Tests restricted to subfields other than entorhinal cortex and subiculum were exploratory only.

## Results

### Participants

In the current study, 66 participants ≥48 years of age were recruited. For 45 of our participants, 2 year follow-up data were available. There were no differences in sex composition (*p* = 0.42), age (*p* = 0.95), education (*p* = 0.42), or MMSE score (*p* = 0.31) between our larger baseline group and the subset with longitudinal data (Table 1). In order to ensure that there were no confounds of age or sex that would make interpreting the GRS signal difficult, we tested for a difference in genetic risk load between men and women (baseline, *p* = 0.82; follow-up, *p* = 0.48), and for a correlation between age and risk score (baseline: *r* = −0.10, *p* = 0.42; follow-up: *r* = 0.01, *p* = 0.94), and detected no significant confounds.

**Table 1:** Cohort characteristics

Characteristic	Baseline participants (*n* = 66)	Follow-up participants (*n* = 45)	*p* value
Sex (M/F)	21/45	18/27	0.421
Age (years; mean±SD)	63.0 ± 10 .4	63.2 ± 7.8	0.953
Education (years; mean±SD)	16.4 ± 2.4	18.0 ± 5.7	0.417
MMSE (mean±SD)	29.2 ± 0.84	28.9 ± 0.86	0.313
Time between visits (years; mean±SD)	N/A	2.12 ± 0.68	N/A

MMSE = Mini Mental State Exam.

### Genetic risk scores

**Figure 3.**
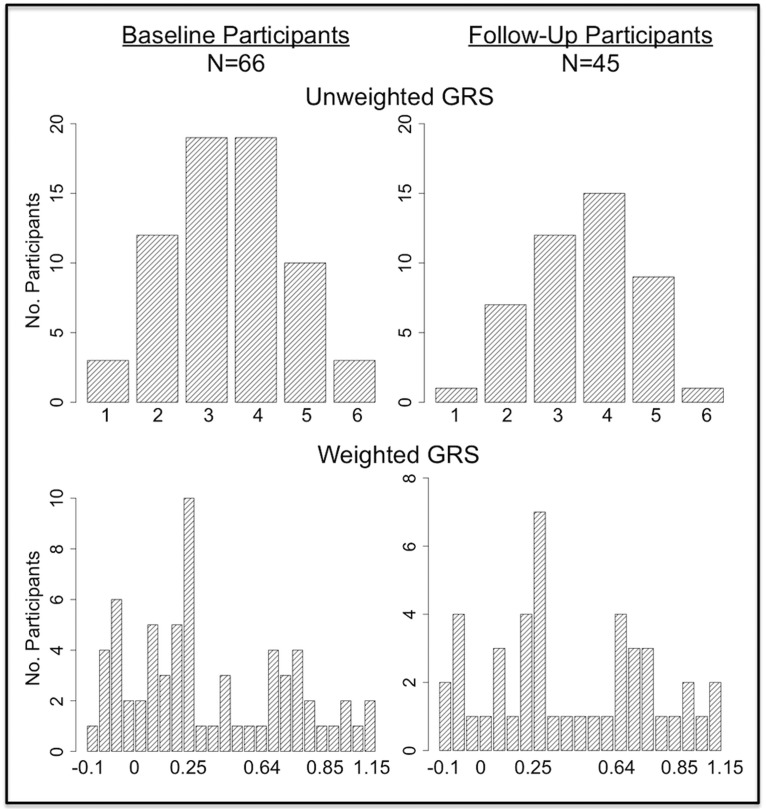
Distribution of genetic risk scores. Participants’ risk scores were normally distributed across the range of possible scores. No participants had zero genetic risk factors nor did any have the maximum of seven risk factors. There were no differences in either unweighted risk score or weighted risk score distributions between the baseline cohort (*n* = 66) and the subset of participants with longitudinal data (*n* = 45).

In our cohort, the URS ranged from 1.0 to 6.0 (Fig. 3). No participant had zero risk factors or the maximum URS of 7.0. WRSs ranged from −0.09 to 1.15 (Fig. 3). As expected, there was a high correspondence between URS and WRS within subjects (*r* = 0.72, *p* < 0.0001). The distributions of risk scores between our baseline group and the follow-up group were not significantly different. We included the WRS in our analyses for transparency, so the effect of weighting could be fairly assessed alongside the additive URS approach. Our focus, however, was on the URS, as this score is most easily and reliably reproduced across research sites.

We tested for an association of verbal memory scores (logical memory delay total and delay total change over 2 years) with GRS, and found no significant relationship between behavior and URS (baseline: *r* = 0.14, *p* = 0.13; follow-up: *r* = −0.06, *p* = 0.34) or WRS (baseline: *r* = −0.06, *p* = 0.34; follow-up: *r* = 0.05, *p* = 0.37). The lack of an association between cognition and genetic risk score highlights the preclinical focus of this work, which is to identify biomarkers that are associated with genetic risk for AD in cognitively healthy older adults.

### High-resolution hippocampal structural imaging: baseline

**Figure 4.**
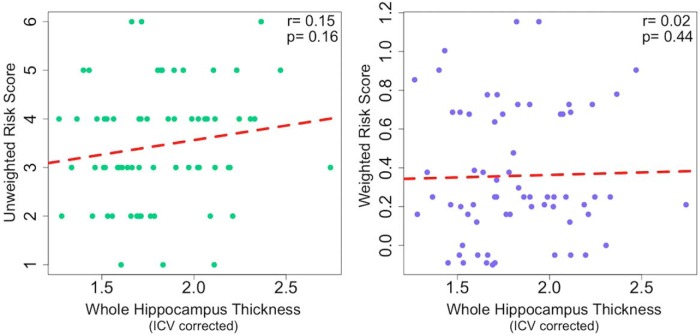
Baseline scatterplots show no association between genetic risk scores and hippocampal complex thickness. Baseline whole hippocampal complex thickness estimates were corrected for normal variation in size using intracranial volume (ICV). There was no significant correlation between unweighted or weighted risk scores and ICV-corrected whole hippocampal complex thickness in our baseline cohort of 66 cognitively healthy older adults.

Baseline HC subfield thickness was corrected for overall differences in size by normalizing each participant’s thickness values by their ICV. There was no significant relationship between GRS and ICV-normalized thickness across the entire HC (URS: *r* = 0.15, *p* = 0.16; WRS: *r* = 0.02, *p* = 0.44; Fig. 4). Next, we examined ERC and SUB, two regions affected early in AD, and again found no association between GRS and ICV-normalized thickness (URS: *r* = 0.14, *p* = 0.13; WRS: *r* = 0.05, *p* = 0.35). As an exploratory analysis, we examined the remaining subfields and did not find any significant relationship between thickness and genetic risk. Finally, we ran partial correlations controlling for the effects of age and sex in the whole HC and in each subregion individually. These partial correlations again showed no significant association between thickness and genetic risk.

### High-resolution hippocampal structural imaging: longitudinal change

**Figure 5.**
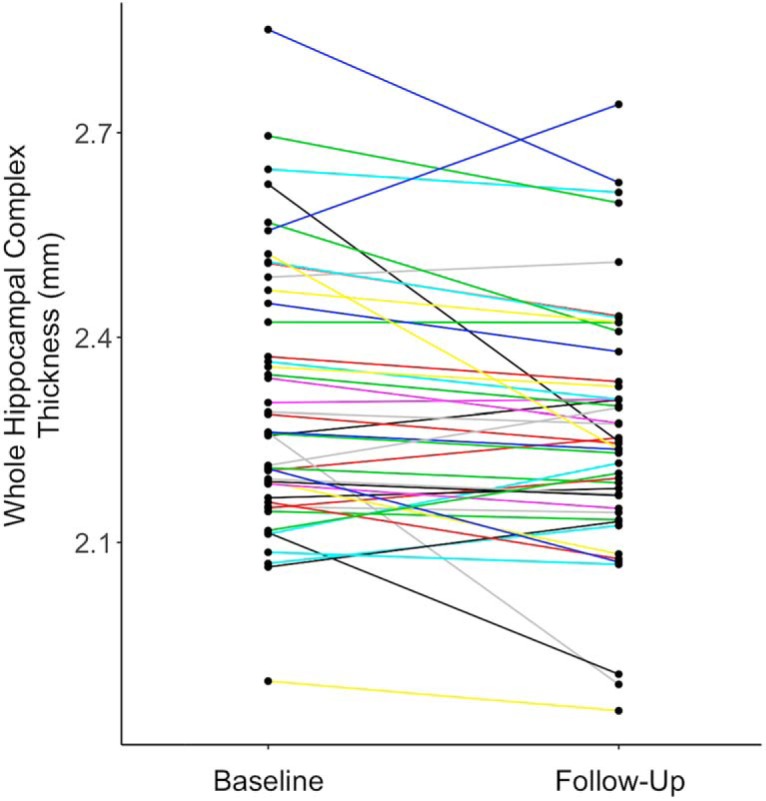
Longitudinal hippocampal complex gray matter thickness. Each participant’s mean thickness across the whole hippocampal complex is plotted at baseline and at follow-up. Most participants experienced modest changes in thickness, while fewer subjects had more dramatic changes in thickness, usually as decreases in thickness. Only one subject had an increase in mean thickness >0.15 mm.

Across the entire cohort, the average change in whole HC thickness was −1.91% (±4.7%) over an average of 2.13 years (±0.68 years). This is slightly higher than previously published estimates of hippocampal atrophy using volumes estimates, but we are using a more sensitive technique, and we include perihippocampal cortical regions in our whole HC average (Das et al., 2012; Fraser et al., 2015). Individual trajectories varied relatively widely, accounting for the large SD in the percentage change in thickness. Most people experienced mild changes in thickness, but a subset had more dramatic changes, usually thinning over time (Fig. 5). There were some individuals whose thickness increased between baseline and follow-up.

**Figure 6.**
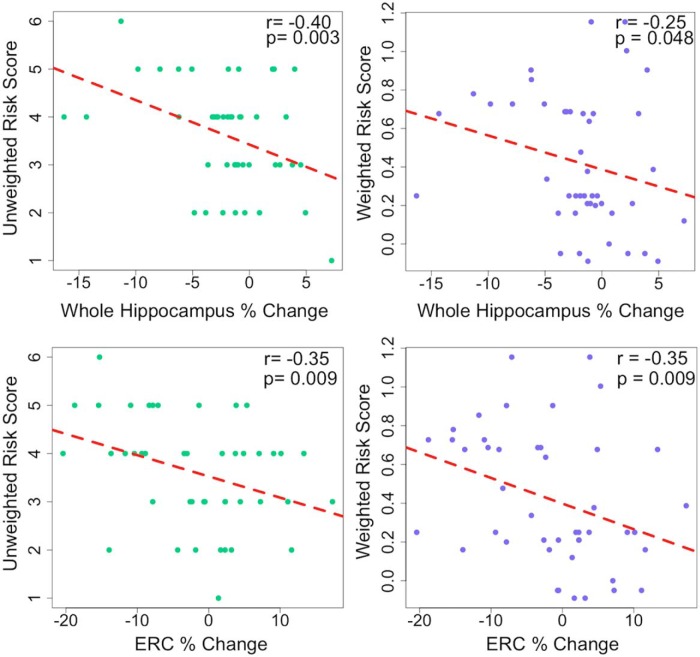
Greater genetic risk score predicts thinning across the hippocampal complex and especially in entorhinal cortex (ERC). There is a significant relationship between both weighted and unweighted risk scores and the percentage change in bilateral hippocampal complex thickness over 2 years. This effect was particularly strong in ERC, a region adjacent to the anterior portion of the hippocampus proper that is affected early in the course of AD.

**Figure 7.**
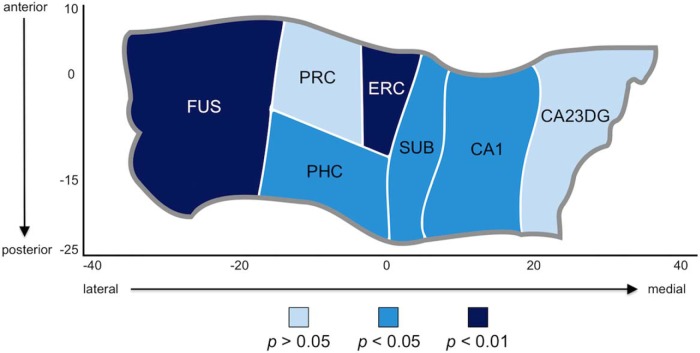
Hippocampal complex unfolded to reveal region-wise relationships to unweighted genetic risk score. A cortical unfolding procedure is used to produce a flat map of the hippocampal complex. Regions are colored according to the statistical strength of the association between unweighted risk score (URS) and the percentage change in thickness between baseline and follow-up scans. In addition to ERC, the fusiform (FUS) showed a significant relationship to URS at *p* < 0.01. Parahippocampal cortex (PHC), subiculum (SUB), and CA1 all showed a significant relationship to URS at *p* < 0.05. The only regions in the hippocampal complex where change in thickness was not associated to genetic risk were CA23 and dentate gyrus (CA23DG) and perirhinal cortex (PRC).

We found a significant negative correlation between increasing GRS and more negative percentage change in cortical thickness across the entire HC (URS: *r* = −0.40, *p* = 0.003; WRS: *r* = −0.25, *p* = 0.048; Fig. 6). We hypothesized that this effect was driven by ERC and SUB, two regions of the HC that are affected early by AD pathology. In ERC, thickness correlated with both GRS types (URS: *r* = −0.35, *p* = 0.009; WRS: *r* = −0.35, *p* = 0.009; Fig. 6). In SUB, the association was significant but not as strong (URS: *r* = −0.31, *p* = 0.01; WRS: *r* = −0.22, *p* = 0.07). We also ran partial correlations controlling for the effects of age, sex, and time between baseline and follow-up scans. Partial correlation coefficients were still significant for whole HC cortical thickness and URS (URS: *r* = −0.34, *p* = 0.028; WRS: *r* = −0.27 *p* = 0.086), and for ERC thickness with both risk scores (URS: *r* = −0.32 *p* = 0.038; WRS: *r* = −0.34 *p* = 0.025). As exploratory analyses, we examined each remaining HC subfield and found additional significant relationships to URS with FUS (*r* = −0.35, *p* = 0.009), PHC (*r* = −0.26, *p* = 0.042), and CA1 (*r* = −0.25, *p* = 0.048) thickness (Fig. 7). Finally, we compared a multiple regression model using our URS to predict a change in whole HC thickness to a model that included only *APOE* as the genetic risk regressor (homozygous carrier = 2, heterozygous carrier = 1, noncarrier = 0; Table 2). Age, sex, and time between baseline and follow-up visits were included in both models. We found that the URS model overall was highly significant (*p* < 0.001) and that URS was a significant predictor within the model (*p* = 0.028), along with time between visits (*p* = 0.002) and a trend for sex (*p* = 0.059). In contrast, the *APOE*-alone overall model was significant (*p* = 0.003), but *APOE* itself was not a significant predictor of thickness (*p* = 0.15). Instead, the model was driven by the time between visits (*p* = 0.002) and sex (*p* = 0.004; Table 2). We used Akaike information criterion (AIC) and Bayesian information criterion (BIC) to directly compare models. Comparing the URS model to the model with *APOE* alone reveals that the URS model is a better fit to our data (URS model: AIC = 258.0, BIC = 268.9; *APOE* model: AIC = 261.2, BIC = 272.1). The URS model was also a better fit when compared with a model that used an FH of AD to quantify genetic risk (URS model: AIC = 258.0, BIC = 268.9; FH model: AIC = 263.0, BIC = 273.9).

**Table 2:** Multivariate models predicting percentage change in hippocampal complex thickness

	Predictors	Coefficients				*R*^2^
		β	SE	*t* value	*p* value	
						
Model 1: unweighted risk score	Constant	17.153	5.994	2.862	0.007**	
	Age	−0.102	0.079	1.291	0.204	
	Sex	−2.730	1.409	−1.938	0.060	
	Years between visits	−2.863	0.893	−3.207	0.003**	
	Unweighted risk score	−1.322	0.583	−2.270	0.028*	
					**<0.001**	**0.364**
Model 2: weighted risk score	Constant	17.032	6.144	2.772	0.008**	
	Age	−0.144	0.079	−1.826	0.075	
	Sex	−3.735	1.337	−2.793	0.008**	
	Years between visits	−2.901	0.915	−3.172	0.003**	
	Weighted risk score	−3.101	1.763	−1.758	0.086	
					**0.002**	**0.333**
Model 3: APOE	Constant	17.227	6.255	2.754	0.009**	
	Age	−0.150	0.080	−1.872	0.068	
	Sex	−4.080	1.349	−3.024	0.004**	
	Years between visits	−2.981	0.932	−3.199	0.003**	
	APOE	−1.249	0.869	−1.437	0.158	
					**0.003**	**0.317**
Model 4: family history	Constant	15.945	6.310	2.527	0.016*	
	Age	−0.146	0.082	−1.782	0.082	
	Sex	−3.702	1.435	−2.579	0.014*	
	Years between visits	−2.699	0.960	−2.810	0.008**	
	FH	−0.895	1.404	−0.637	0.528	
					**0.007**	**0.289**

**p<0.01

*p<0.05

## Discussion

We have shown that a GRS for AD is associated with hippocampal thinning over 2 years, but not with baseline morphology, in cognitively healthy older adults. Our findings provide evidence that genetic risk screening might be a valuable tool for predicting the trajectories of endophenotypes and, ultimately, disease. By showing that greater genetic risk is associated with greater thinning in the hippocampus, a region that is particularly vulnerable to AD pathology, we demonstrate the power of working with a neuroimaging genetics approach in cognitively healthy individuals. There were no associations between GRS and baseline hippocampal morphology, which was not in line with our hypotheses. This highlights the importance of longitudinal data, especially when studying healthy older volunteers, for identifying differences in atrophy rates which are likely more sensitive than baseline differences (Lu et al., 2011; Chincarini et al., 2016). There were also no associations between GRS and verbal memory performance in our participants, which supports the idea that neuroimaging endophenotypes for AD may be more sensitive markers of risk for disease progression during the preclinical phase. Our study identified a predictive relationship between genetic risk for AD and hippocampal complex thinning that is not mediated by cognition. Thus, our findings demonstrate a truly preclinical potential biomarker for AD.

Other investigators have taken polygenic AD risk score approaches in neuroimaging studies. One of the first of these studies reported that a GRS that included all of the then known AD risk genes predicted cortical thinning in regions that are particularly vulnerable to AD, including entorhinal, lateral temporal, and posterior cingulate cortices (Sabuncu et al., 2012). Another more recent study (Chauhan et al., 2015) used a similar approach, combining all known AD risk genes into a single score, and examined several structural measures in a large cohort of cognitively healthy subjects. The authors found that a higher genetic risk score was significantly associated with hippocampal volume, but not with intracranial volume or whole-brain volume. Our results, like those from these studies, support the existence of a predictive link between genetic risk for AD and hippocampal complex morphology.

The present study design has two unique strengths. The first is the two-level selection criteria that we used in creating our GRS. While there is certainly a defensible rationale for creating risk scores that include every known genetic locus with a significant association with disease, we argue in favor of a hypothesis-driven approach restricted to genes and factors for which evidence links them to the biomarker of interest. It unlikely that every genetic risk factor associated with AD incidence is also significantly associated with a given AD biomarker, such as hippocampal integrity. It is more likely that many of the genetic loci associated with AD incidence have nonoverlapping molecular mechanisms leading to increased risk for disease and, therefore, would likely drive changes in some biomarkers for AD and not others. For example, studies have shown that *CLU* variants are related to structural and functional MRI biomarkers, but no association with positron emission tomography (PET)-measured amyloid deposition or to AD-relevant CSF analytes has been reported. Our approach of using only genetic variants associated with AD incidence and also hippocampal structure or function strengthens our ability to detect a significant association with hippocampal atrophy. A second strength of our study is the process by which we measure our biomarker, hippocampal complex cortical thickness. Volumetric measurements of the hippocampus based on whole-brain structural imaging are less sensitive to subtle changes in gray matter morphology than the semi-manual hippocampal segmentation process with high-resolution, partial-field-of-view imaging used in this work. In preclinical AD, specific cortical laminae experience neuronal loss, which affects total volume only subtly while exerting a greater effect on gray matter thickness measurements (Hyman et al., 1984; Gómez-Isla et al., 1996). Also, cortical thickness measurements are calculated at hundreds of points across the gray matter of the hippocampal complex, making averages more robust and less likely to be influenced by noise or error than lower-resolution volume estimates that include regions of white matter and, sometimes, CSF.

Our method of hippocampal subfield segmentation is one of several such techniques. There is an ongoing effort to create a harmonized protocol for hippocampal subfield segmentation, which we are actively supporting (Boccardi et al., 2011; Yushkevich et al., 2015). These efforts are essential to ensure that findings from different research groups are comparable and, therefore, better serve to enhance our understanding of hippocampal morphology and pathological changes to hippocampal structure. However, our laboratory has been consistently and successfully using versions of our current method for >10 years, and it is the most reliable method available, especially as it pertains to the segmentation of the most anterior hippocampal subfields, including entorhinal cortex (Ekstrom et al., 2009; Donix et al., 2010a; Burggren et al., 2011). In future studies, we plan to adopt the automated techniques resulting from the Hippocampal Subfields Working Group efforts. In the present study, we chose not to interrogate left and right hippocampal complexes separately because we did not have a hypothesis regarding the laterality of the association between an AD GRS and hippocampal thinning.

We recognize that the factors included in our genetic risk score are not entirely independent. For example, carriers of the APOEε4 allele often have a higher incidence of positive family history of AD when compared with APOEε4 noncarriers (Zintl et al., 2009). However, due to our recruitment strategy targeting the worried well and older adults with a family history of AD, APOEε4 noncarriers in our cohort are enriched for other genetic risk factors for AD, such as family history of AD, despite their lack of an APOEε4 allele. In our cohort, there were no significant differences in family history in carriers (60.7% with positive family history) versus noncarriers (65.8%) of the APOEε4 allele (*p* = 0.80).

There are several ways to attempt to identify genetic risk factors associated with a particular endophenotype, including data reduction techniques such as principal component analysis and regression techniques such as logistic regression in genome-wide association studies (GWASs). In the present study, we chose to use the two-level selection criteria approach due to its conceptual novelty. Our use of an OR-weighted GRS and an unweighted GRS side by side was meant to illustrate the advantage of one over the other, if present. However, we found that in our GRS, composed of four genetic risk factors, it was at least equally effective to use a simple linear additive risk score as it was to use a weighted approach. Because odds ratios change slightly with each GWAS, a simple additive approach might be best to ensure comparability and reliability of a GRS across laboratories and in clinical trials.

In addition to hippocampal integrity, another potential biomarker of preclinical AD is amyloid and tau deposition, as measured by PET scanning. We do not have amyloid- or tau-PET data available on these subjects, so it is not possible to rule out the presence of these pathologies in these subjects. We are also not able to estimate the tau positivity rate based on the literature as tau-PET scanning is a relatively new tool, but there is evidence that, like amyloid, tau is sometimes present in high levels in the brains of clinically healthy individuals (Johnson et al., 2015). According to Doraiswamy et al. (2014), ∼14% of cognitively healthy older subjects are amyloid positive. Of course, the cutoff to define amyloid positivity is not precise and varies across studies, so this positivity rate is just an estimate. Still, assuming that this rate is accurate, it would indicate that 9–10 (9.24) participants in our cohort are likely to be amyloid positive. Thus, we feel that even with the potential noise introduced into our sample by possibly including subjects with amyloid, we still have a large enough sample of amyloid-negative participants to detect the significant effect between GRS and hippocampal morphology.

Mechanistic insights from neuroimaging genetics studies are inherently limited by the lack of known causal variants driving many of the significant GWAS signals in AD. Saykin et al. (2015) describe a multistep process to move from these genetic signals to targeted therapeutic agents. In their model, genetics and neuroimaging intersect at the first step (discovering genetic loci that are robustly associated with a relevant trait) and at the final step (identifying individuals most likely to benefit from experimental therapies). The steps linking these two together include, first, the identification of causal variants, then testing hypothesized mechanisms in model systems, and, finally, developing mechanism-targeted therapeutic agents. In the present study, we have demonstrated an additive effect of multiple genetic risk factors on an AD biomarker, indicating that there might be different mechanisms affecting the same outcome measure, in this case hippocampal complex cortical thickness.

The present study provides the first evidence that a hypothesis-driven AD GRS predicts increased hippocampal complex subfield thinning over 2 years in healthy older adults. This work is extremely relevant to clinical trial design because of the short, 2 year follow-up time along with the ease of collecting genetic and MRI data. Both are minimally invasive and can be repeated as needed. We argue that prescreening preclinical, cognitively healthy individuals to maximize genetic risk will increase the power to detect changes in related biomarkers. Indeed, preliminary work designed to assess the increased power of genetic prescreening in clinically impaired cohorts has been promising. Kohannim et al. (2013) report up to a 50% decrease in sample size needed to detect an effect in atrophy over 2 years. Genetic prescreening paired with neuroimaging-based outcome measures is going to be a critical component of future AD clinical trials focused on cognitively healthy, preclinical individuals for which traditional pencil-and-paper outcome measures will not be sensitive enough to detect drug effects.

## Synthesis

The decision was a result of the Reviewing Editor Howard Eichenbaum and the peer reviewers coming together and discussing their recommendations until a consensus was reached. A fact-based synthesis statement explaining their decision and outlining what is needed to prepare a revision is listed below. The following reviewer(s) agreed to reveal their identity: Christian Habeck

The reviewers are largely satisfied with the revisions, but would like you to respond to a remaining concern by one reviewer:

There is one slight point of confusion, possibly brought on by myself. By "truly multivariate" analysis, I did not mean to suggest adding isolated loci as independent variables in a multivariate linear regression. Rather, I meant employing some form of multivariate decomposition for data reduction and fashioning of independent variable for predicting of thinning as an outcome measure; one could see this as the simplest form of a Machine-Learning approach. For instance, do PCA on the whole array of loci, then some subset selection (with AIC, BIC, or cross-validation) do pick particular PCs to be used for predicting cortical thinning, leading to a genetic risk profile derived de-novo, rather than taking univariate log-odds as the weighting coefficients for a linear-combination. In my anecdotal experience, genetic analysts are loath to perform PCA or similar decompositions because they think of these tools as mainly identifying nuisance variability (ethnicity etc.) On the other hand, I don't deal with genetic data regularly and my rendition here might have missed something crucial and a definite conclusion on these matters might not have been reached yet. All these issues would be very suitable for discussion, even if some consensus is still outstanding...
